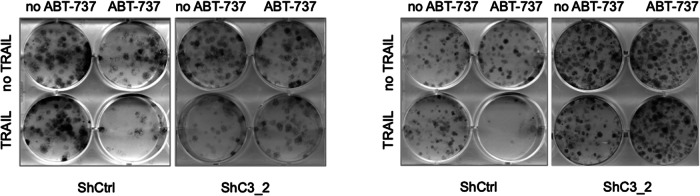# Correction: ABT-737 promotes tBid mitochondrial accumulation to enhance TRAIL-induced apoptosis in glioblastoma cells

**DOI:** 10.1038/s41419-024-06649-y

**Published:** 2024-09-17

**Authors:** S. Cristofanon, S. Fulda

**Affiliations:** 1 Institute for Experimental Cancer Research in Pediatrics, Goethe-University, Frankfurt, Germany; 2 University Children’s Hospital, Ulm, Germany

Correction to: *Cell Death and Disease* 10.1038/cddis.2012.163, published online 29 November 2012

Since online publication of this article, the authors noticed that Fig. 4c does not show the correct blots for U118MG cells. The correct Fig. 4c that was also originally submitted to the journal for review of the manuscript is provided below. This unintentional mistake does not alter the conclusions of the study.
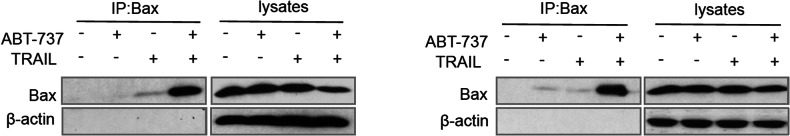


Figure 6d does not show the correct colony assays for U87MG and U118MG cells transduced with a vector containing shRNA sequence against caspase-3 (ShC3_2). The corrected Fig. 6d is provided below. This unintentional mistake does not alter the conclusions of the study. The authors apologise for any inconvenience caused.